# Works of Hippocrates and Galen

**Published:** 1846-02-01

**Authors:** 


*Works of Hippocrates and Galen.*—Dr. John Redman Coxe, of Philadelphia, formerly Professor of Chemistry in the University of Pennsylvania, and distinguished especially for his medical antiquarian lore, proposes to publish a translated epitome of the above works if 500 subscribers can be obtained. It will consist of two or three volumes of from 500 to 600 pages each, at a price not exceeding $3 00 a volume. Physicians wishing to subscribe can forward their names to Lindsay & Blakiston, Philadelphia.

